# Connectivity Analysis Using Functional Brain Networks to Evaluate Cognitive Activity during 3D Modelling

**DOI:** 10.3390/brainsci9020024

**Published:** 2019-01-24

**Authors:** Muhammad Zeeshan Baig, Manolya Kavakli

**Affiliations:** Department of Computing, Faculty of Science and Engineering, Macquaire University, Sydney, NSW 2109, Australia; manolya.kavakli@mq.edu.au

**Keywords:** novice, expert, transfer entropy, information flow pattern, functional brain network, 3D modelling

## Abstract

Modelling 3D objects in CAD software requires special skills which require a novice user to undergo a series of training exercises to obtain. To minimize the training time for a novice user, the user-dependent factors must be studied. we have presented a comparative analysis of novice/expert information flow patterns. We have used Normalized Transfer Entropy (NTE) and Electroencephalogram (EEG) to investigate the differences. The experiment was divided into three cognitive states i.e., rest, drawing, and manipulation. We applied classification algorithms on NTE matrices and graph theory measures to see the effectiveness of NTE. The results revealed that the experts show approximately the same cognitive activation in drawing and manipulation states, whereas for novices the brain activation is more in manipulation state than drawing state. The hemisphere- and lobe-wise analysis showed that expert users have developed an ability to control the information flow in various brain regions. On the other hand, novice users have shown a continuous increase in information flow activity in almost all regions when doing drawing and manipulation tasks. A classification accuracy of more than 90% was achieved with a simple K-nearest neighbors (k-NN) to classify novice and expert users. The results showed that the proposed technique can be used to develop adaptive 3D modelling systems.

## 1. Introduction

Presently, 3D technology fascinates everyone with skillful use of 3D computer graphics and special effects in movies, video games, and visual arts. People are highly motivated to construct their own 3D objects using their imagination; however, the software packages are still quite complex and require special training. It is difficult for a non-expert, but also for skilled people, because these CAD tools require time and skills to learn the necessary commands and techniques to draw. How can we minimize the time and skills required to make CAD packages accessible for a common user? This question motivates us to investigate the differences in information processing in users using simplified EEG headsets.

Differences in information processing have been observed between males and females, novices and experts, and left-handed and right-handed people while describing a simple 3D object [1]. The study of these user-dependent factors makes the interaction robust, and enhances system flexibility, efficiency, naturalness. The purpose of this research is to investigate the differences in information flow patterns by analyzing the electroencephalogram (EEG) signals of various participants and to find out the relationships between cognitive strategies in information processing and expertise while performing a set of experiments. The connectivity measures are commonly used in the literature to study user-dependent factors in the brain [2].

An EEG signal contains information from a complex and dense network of billions of interconnected neurons. Graph-based methods have been applied by researchers to successfully study these complex brain networks in recent years [3] and more specifically using both directional and undirected functional brain networks (FBN) [4,5]. In research, directional FBN is preferred because it provides more prominent topological features by estimating the direction of information transfer between nodes (EEG electrodes) and thereby enabling more detailed analysis [6]. To construct an FBN using an EEG, a connectivity measure is used that can estimate the value of information transferred between nodes (electrodes). There are many linear and non-linear connectivity measures that have been used in the literature to construct the FBN, such as mutual information, entropy, correlations, and Granger causality [7]. Linear connectivity measures usually fail to identify the non-linear behavior of the brain. Therefore, to analyze a highly non-linear EEG signal, non-linear measures are adopted by researchers for the construction of FBNs [8].

Transfer Entropy (TE) is also a popular measure to quantify information between two non-linear vectors. It can also determine the direction of information transfer between two variables [9] and therefore is ideal for investigating information flow. TE uses the past activity of both variables to estimate the amount of activity of a system irrespective of interaction model. This property of TE allows researchers to apply it to various applications such as identifying information transfer between auditory cortical data [10], localization of epileptic patients focus [11], the effect of heart rate on breath rate [12], and information flow patterns in various driving states [13]. Other brain connectivity measures such as partial directed coherence (PDC) and directed transfer function (DTF) was used in the literature to evaluate the performance during meditation [14,15].

In research literature, TE has not been used to study user-dependent differences. In the present work, we apply TE to the analysis of information flow patterns between novice and expert users. We have used normalized TE values to construct both binary and weighted directional FBNs. After constructing an FBN, we have applied graph theory measures and statistical analysis to quantify the information flow patterns. The main objective of this analysis is to identify the topological differences between novice and expert users’ FBNs in different design activities. The second objective is to identify the information flow patterns in the design actions of a user.

## 2. Materials and Methods

We have constructed an FBN using Normalize TE as a connectivity measure. The NTE matrix is used for constructing binary and weighted FBN.

### 2.1. Normalized Transfer Entropy

NTE is calculated by dividing the difference of TE matrix from a shuffled version of that TE matrix by conditional entropy. The subtraction of shuffled TE overcomes the noise/bias of TE generated due to the finite and non-stationary data [16,17]. To calculate NTE, we calculated TE given in Equation (1):

(1) $$TE_{y\to x}=\sum_{x_{n+1},x_{n},y_{n}}p\left(x_{n+1},x_{n},y_{n}\right)log\left(\frac{p\left(x_{n+1},x_{n},y_{n}\right).p\left(x_{n}\right)}{p\left(x_{n},y_{n}\right).p\left(x_{n+1},x_{n}\right)}\right)$$

where, $x_{n}$ is the value of signal *x* at time *n*, $y_{n}$ is the value of signal *y* at time *n*, and p(.) is the probability distribution. NTE from y→x is calculated with respect to the total information in *x* and represent the relative amount of information transferred by signal *y*. The equation for calculating NTE matrix from a vector y→x is given in Equation (2) as:

(2) $$NTE_{y\to x}=\frac{TE_{y\to x}-<TE_{y_{shuffle}\to x}>}{H(x_{n+1}|x_{n})}$$

where $TE_{y\to x}$ is the transfer entropy from y to x and can be calculated using Equation (1), $<TE_{y_{shuffle}\to x}>$ is shuffled TE from *y* to *x* using shuffled version of *y* and $H(x_{n+1}|x_{n})$ is the conditional entropy of *x* at time n+1 given its value at time *n* and calculated as given in Equation (3). In Equation (2), $y_{shuffle}$ contains the symbols that are rearranged and shuffled in random order.

(3) $$H\left(x_{n+1}|x_{n}\right)=-\sum_{x_{n+1},x_{n}}p\left(x_{n+1},x_{n}\right)log\left(\frac{p(x_{n+1},x_{n})}{p(x_{n})}\right)$$

The NTE from y→x is not equal to NTE from x→y and NTE is in the range of 0 and 1. If the value of NTE is 0 that means no transfer of information and if the value is 1 than the information transfer is maximum [13].

### 2.2. Functional Brain Networks Graph Theoretical Analysis

FBNs are used to capture the information flow dynamics between EEG electrodes in relation to the human brain [18]. EEG is a common tool to measure brain activity as it is inexpensive, non-invasive, and has high temporal resolution. EEG has been used in many applications ranging from medical diagnostics, cognition, to brain–computer interfaces [19,20,21]. In this paper, FBNs were constructed from a non-linear connectivity measure known as NTE. After constructing FBNs, graphs were constructed by considering each electrode as a node and the NTE value as a link.

A graph is a combination of nodes and links. Nodes are vertices, and links are the edges that are used to connect the vertices. In our case, the nodes are electrodes and the links are established by the connectivity measure NTE [22]. The following graph theory measures are used to analyze an FBN.

#### 2.2.1. Connectivity Density

Connectivity density (CD) is the ratio of the actual number of edges to the total number of possible edges [23]. The value of the CD is in the range of 0 and 1. If a graph has complete connectivity then it has a CD of 1.

#### 2.2.2. Motif

A motif is used to describe the local structure of a graph. It is the patterns of interconnections that can occur in a complex network [24]. It is further characterized by the number of times a subgraph appears in a complex network [25]. In the case of 3 nodes, a total of 13 classes of subgraph can be generated. In this research, we have considered 3 nodes for motif count.

#### 2.2.3. Node Strength

Node strength is used to measure the centrality of weighted directed networks. It represents the sum of all incoming and outgoing edge weights [26]. Node strength helps to find the involvement of a particular region in an FBN.

#### 2.2.4. Small-Worldness

The small-world network exists in between regular lattice and completely random network and shows the properties of high clustering and short path length, which means that information traverse mostly between nodes with a small number of edges not between neighbors [27]. It is also considered as a network with both high local and global efficiency [28] which is a representation of effective information propagation over a network.

##### Clustering Coefficient

The clustering coefficient is the ratio between all the directed triangles formed by node *i* and the number of all possible edges node *i* can form, so the clustering coefficient measures how well the cluster of node communicating and a high value of clustering coefficient relates to the high local efficiency of information transfer [4].

##### Characteristic Path Length

The characteristic path length is the average shortest path length between all pairs of nodes. It indicates the high global efficiency of information transfer. The shortest path between node *i* and *j* is the minimum number of nodes to traverse from node *i* to reach to node *j* [4].

##### Small-World Index

Small-world index gives us the comparison of a complex network to a random network. A random network has a low clustering coefficient and typically a short path length compared to a complex network [29]. The small-world networks have high global and local efficiency and categorized as those networks whose small-world index σ>1 [30].

All above-mentioned graph theory measures have been used for in this analysis.

## 3. Methodology

### 3.1. Experimental Setup and Data Acquisition

The experiment was to design a 3D table with three parts: a base, a pillar, and a top in AutoCAD. The next task was to use properties and options to change the object’s colors and add materials. A total of eight participants volunteered for the experiment. All of them were computer-science students at Macquarie University. The ages of the participants ranged from 21 to 30 years. Three of the participants had prior knowledge of AutoCAD and 5 were novices. The experiment was approved (Approval no. 5201700784) by the Faculty of Science and Engineering Human Research Ethics Sub-Committee, Macquarie University. This research meets the requirements of the National Statement on Ethical Conduct in Human Research (2007). The National Statement is available at the following web site: http://www.nhmrc.gov.au/_files_nhmrc/publications/attachments/e72.pdf.

**Figure 1:**
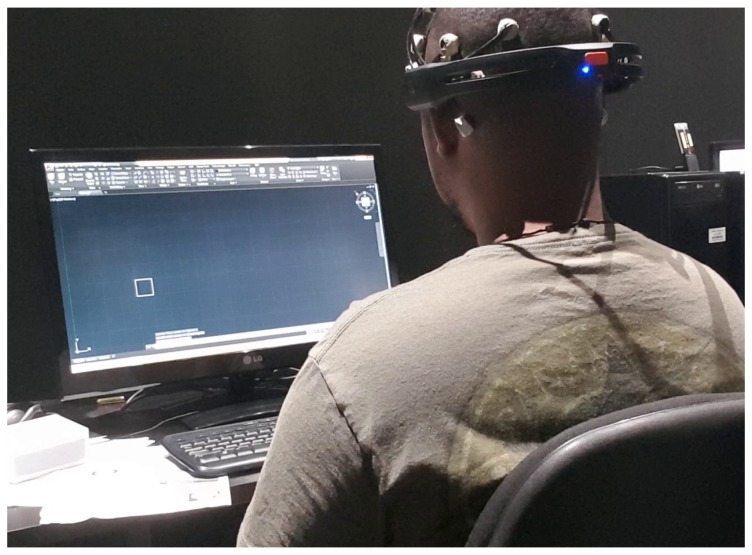
A subject is using AutoCAD and wearing EEG headset in real experimental setting.

Each subject was given a tutorial of 10 min before the experiment. A video log has been maintained for each subject and EEG signals were also recorded. For acquisition of EEG signals, we used an off-the-shelf research edition of the Emotiv EEG headset, which has 14 channels: frontal and front-central: AF3, AF4, F3, F4, F7, F8, FC5, FC6; temporal: T7, T8; and the occipital and occipital-parietal: O1, O2, P7, P8. The device has an internal sampling rate of 2048 Hz which is down-sampled to 128 Hz after the cleaning of artifacts. The electrode placement is based on the international 10-20 system. The problem with the Emotiv Headset is that it has limited electrodes with no electrodes in the central lobe (Pz, Cz, Fz) and therefore, it is thought that the system has limited applicability in research. The manufacturer states that the signals from the neighboring electrodes are good enough to perform experiments and researchers have proved the capability of headset in many applications [31]. The subjects were given an open-ended task to depict the real-world setting, which results in complex cognitive processing and analysis strategies. A picture of the experimental setup is shown in Figure 1.

### 3.2. Experimental Procedures

The process can be divided into 5 experimental procedures:

1. Information about the experiment was given to each subject along with the consent form. After reading and signing the consent form, the experimenter gives a walk-through of the experiment and some instructions to minimize the body and head movements.
2. The EEG headset was placed on the head of the subject. The subjects were asked to rest for two minutes with their eyes open, with hands on their laps, and after that, the subjects were asked to start drawing using keyboard and mouse.
3. After the completion of the modelling task, subjects were asked to fill in a questionnaire about the experiment.
4. EEG data were filtered and segmented into two segments. The first segment was the one where the participants were drawing the table. In the second segment, the participants were asked to change the appearance of the table such as materials, colors etc. using AutoCAD options. For convenience, we named the first segment “Drawing” and the second segment “Manipulation”.

### 3.3. EEG Signal Pre-Processing

The EEG data of all the participants were used for the analysis. The pre-processing was done in MATLAB 2017b using the EEGLAB toolbox [32]. The baseline was removed from the EEG signal and low-pass filtering at a cut-off frequency of 45 Hz was performed using a linear-phase FIR filter. EEG signals were then high-pass filtered at a cut-off frequency of 0.1 Hz and notch filtered at 50 and 60 Hz using a linear-phase FIR filter. The order of the filter in all cases was 300. After filtering the data, the typical eye blinking and muscles artifacts were removed manually by visual inspection. To estimate the position of the artifacts, we have used the runica ICA decomposition. This method decomposes EEG signals into their independent components. Further noisy bad blocks were also removed manually. Once the data was clean enough, we extracted two-second epoch averaged data from resting, drawing and manipulation tasks by performing back-to-back epoching with a 0.5 s difference between epochs. The response time for a modelling action varies from 0.5 to 4 s and we made sure that a minimum of one modelling action must be performed in an epoch through the video log. Table 1 shows the time taken by each user in seconds in drawing and manipulation states. The users 1, 7 and 8 were expert in AutoCAD, whereas, users 2–6 were novices. User 3 and 7 both deleted some objects when drawing and these phases were also considered in this experiment. There was no correlation found between expertise level and task completion time.

### 3.4. Functional Brain Network

**Figure 2:**
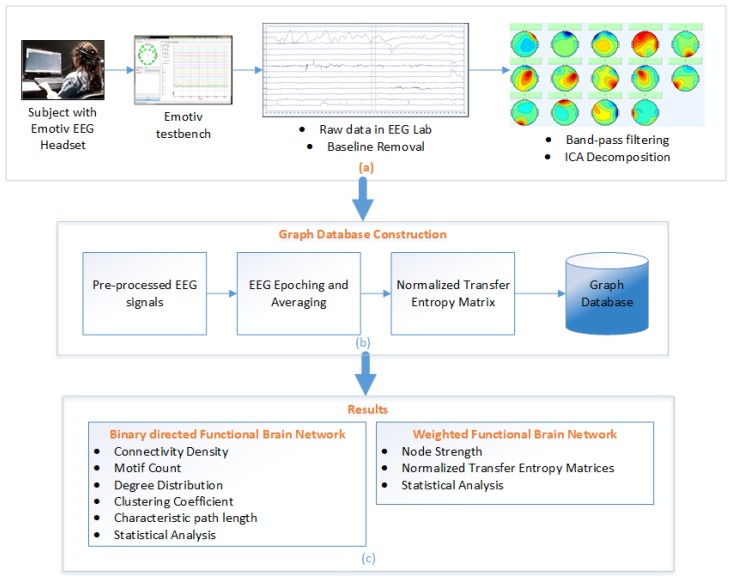
Normalized Transfer Entropy Framework (**a**) EEG data acquisition and pre-processing, (**b**) Transfer Entropy calculation and graph database construction, and (**c**) Result and analysis of binary and weighted FBNs.

The pre-processed EEG signals were used in the construction of NTE connectivity matrices, where each cell denotes the NTE value from one electrode to another. The normalization was done by subtracting a noise matrix (averaged shuffled TE matrix) from the TE matrix. The NTE matrices were used to create both binary and weighted directed FBN. To analyze the results, we had used the graph analysis measure such as the CD, clustering coefficient, characteristic path length, motif count, node strength and small-worldness. The Figure 2 shows a summarized view of the experiment.

### 3.5. Binary Directed Functional Brain Network

A threshold was applied on NTE matrix to convert them into binary directed FBN for calculating complex network parameters. For this experiment, the threshold was set to 0.001 which is an arbitrary value to remove the very insignificant connections.

### 3.6. Weighted Directed Functional Brain Network

**Figure 3:**
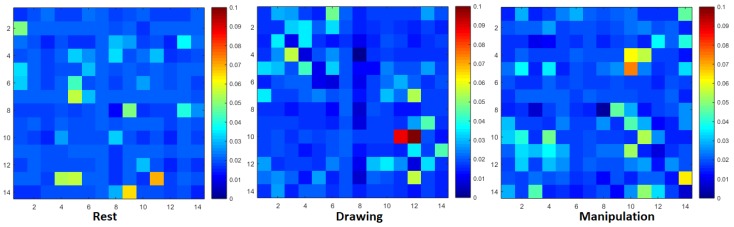
Normalized transfer entropy matrix during rest, drawing and manipulation states of novice user.

**Figure 4:**
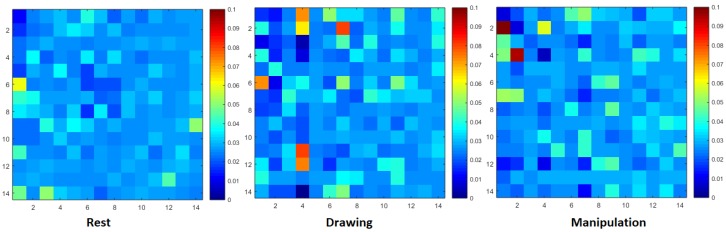
Normalized transfer entropy matrix during rest, drawing and manipulation states of expert user.

The NTE matrices, without applying any threshold, shown in Figure 3 and Figure 4, were also used to design weighted directed functional brain networks (WDFBN). WDFBN was used to calculate the node strength using Equation (4).

(4) $$Strength_{i}=\sum_{j\epsilon V}w_{ij}+\sum_{J\epsilon V}w_{ji}$$

where, $w_{ij}$ is an element weight of NTE matrix.

#### 3.6.1. Hemisphere-Wise Information Flow

**Figure 5:**
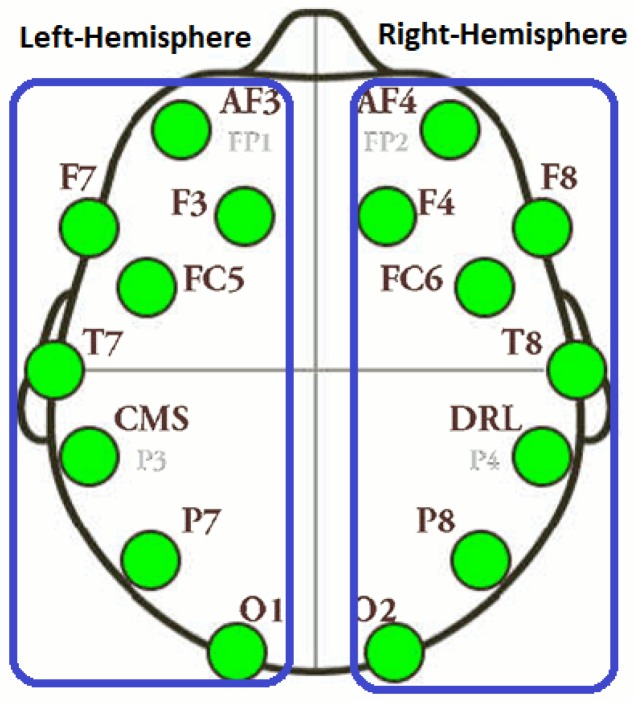
Electrodes layout of LH and RH.

The hemisphere analysis has been performed to identify the information flow patterns within and between the left and right hemispheres. For this analysis, we have divided 14 EEG electrodes into two sets of 7 electrodes that correspond to each hemisphere. The electrodes belong to left hemisphere (LH) and right hemisphere (RH) are shown in Figure 5.

A total of four sub-NTE matrices were generated to represent the information flow between electrodes in LH to LH, LH to RH, RH to RH and RH to LH. The size of sub-NTE matrices was 7 × 7 and the total information flow from one electrode to all other electrodes was calculated by row-wise summation of each sub-NTE matrices. To analyze these sub-NTE matrices, one-way ANOVA was applied and the results of 2 typical novice and 2 typical expert users have been shown in this paper due to space limitations.

#### 3.6.2. Region-Based Information Flow

**Figure 6:**
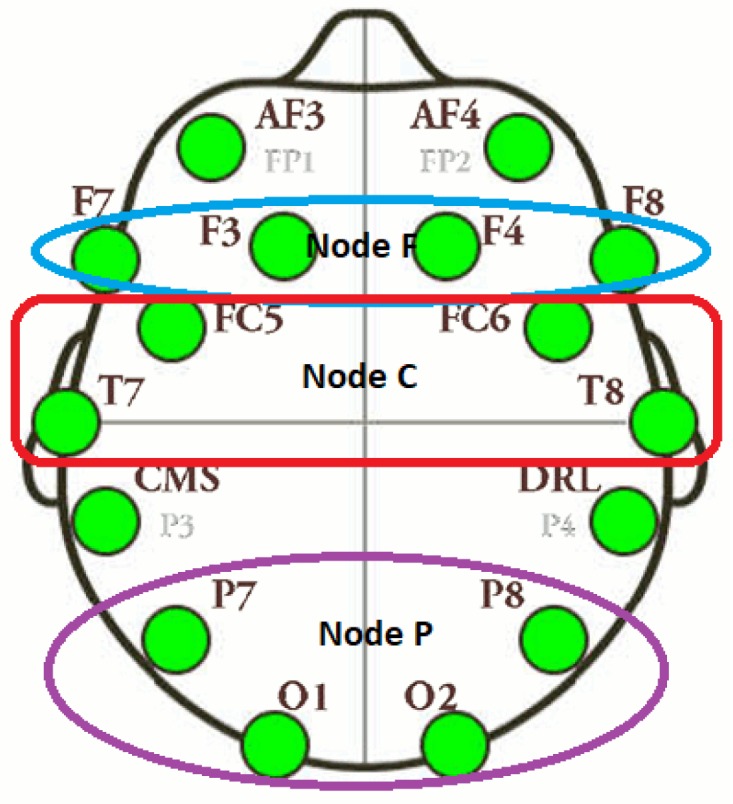
Electrodes layout of F, C and P nodes.

To study the information flow (IF) in different regions of the brain, we also performed the region-wise analysis. We divided the brain into three regions and called them nodes. Each of the three nodes had four electrodes. From the NTE matrix, three sub-matrices were constructed and information flow from and to the node was calculated. The nodes are F, C, and P. The node F contains electrodes F7, F3, F4 and F8 and represent the frontal cortex of the brain. The node C contains electrodes FC5, T7, T8 and FC6 and represent the central and temporal cortex. The node P contains electrodes P7, O1, O2, and P8 and gives the information of parietal and occipital lobes. Figure 6 shows the three nodes with corresponding electrodes.

The information flow from one region to another region was calculated by the summation of information flow from electrodes of one region to other. For example, the IF from F to C was calculated by summation of all IF from F7, F3, F4, F8 to FC5, T7, T8, FC6.

### 3.7. Classification of Novice/Expert

**Figure 7:**
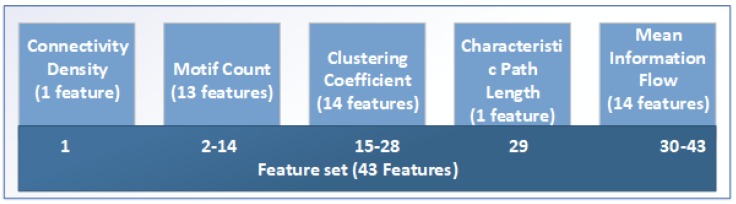
The complete feature set.

We have used the above-mentioned graph-based measures as a feature for the classification of novice and expert users. To depict real-time situations, a 20-s window from every second onward was used and considered as one sample. By this method, we extracted a total of 470 samples in which 285 samples belong to novice users and 185 samples belong to experts. The CD, motif count, clustering coefficient and mean information flow are used as a feature for classification. The complete feature set is the combination of all the measures mentioned above. The actual feature set is given in Figure 7.

Feature selection algorithm was applied to extract best features for classification. We have used a sequential forward search (SFS) for searching the best feature. The technique used for feature selection is wrapper technique. In the wrapper technique, the classification algorithm is a part of the feature selection process. In this paper, classification accuracy has been used as the optimization criteria for feature selection.

Various classification algorithms are used to classify features into different classes. These classification algorithms are categorized into supervised and unsupervised techniques. Support Vector Machines (SVM) [33], K-nearest neighbors (k-NN) [34], Bayesian classifier [35] are some of the most commonly used classifier for EEG applications. In this paper, we have used five different classifiers to test the feature set. The classifier used in this paper are SVM, k-NN, Linear Discriminant Analysis [36], Naive Bayes [35], and decision trees [37]. For evaluation we have used classification accuracy, sensitivity, specificity, precision, F-measure and mean squared error.

## 4. Results and Discussion

The NTE matrices of size 14 × 14 were calculated as shown in Figure 3 and Figure 4. Figure 3 shows the NTE matrix of a novice user with three different design states and Figure 4 shows the NTE matrix of an expert user. The cluttered and brighter pixel shows the increase in information flow. From the NTE matrices, it can be inferred that the information flow pattern of the user changed and increased from the baseline rest condition. Due to space limitation and maintain the readability of the paper, the results of two novice and two expert users have been shown in this paper instead of the entire cohort. Most of the figures and tables in this section show the results of some particular participants.

### 4.1. Binary Directed FBN Analysis

**Figure 8:**
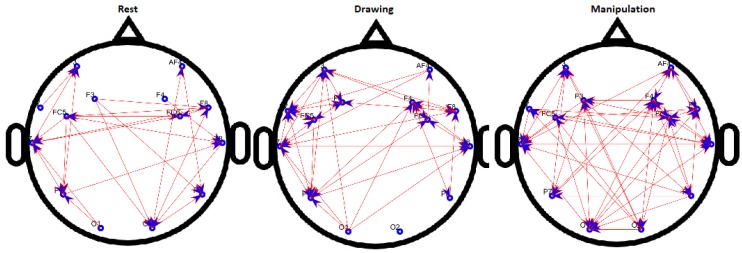
Binary directed functional brain network during rest, drawing and manipulation states of novice user.

**Figure 9:**
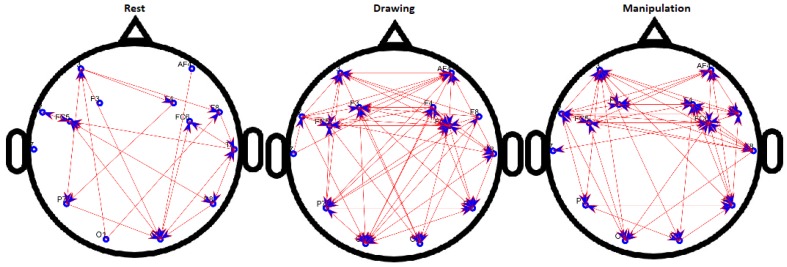
Binary directed functional brain network during rest, drawing and manipulation states of expert user.

The results of binary directed FBNs are shown in Figure 8 for a novice user and Figure 9 for an expert user. For the novice, the connections between electrodes increase when participants move from rest to drawing and from drawing to manipulation. Whereas, connections increase from rest to drawing and no significant change was observed from drawing to manipulation for expert users.

It can be seen that the density of network increased from the baseline for both novice and expert users but the change in density for the novice user is more, compared to an expert user in drawing and manipulations states. Most of the activity was focused on the frontal cortex which also indicates the use of short-term memory [38].

**Figure 10:**
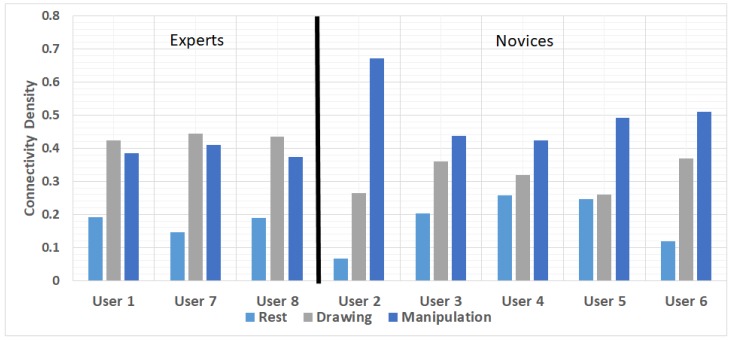
Comparison of connectivity density of all users brain activity during rest, drawing and manipulation states.

The CD for all the user is shown in Figure 10. To compare the CD across all users in different states, we normalized the value by dividing the actual connectivity value in one state by the sum of connectivity in all states.

The users 1, 7 and 8 were experts as they have previous experience in AutoCAD, user 4 and 5 were novices but they had a go with the experiment before as well. As indicated in Figure 10, the CD is higher for all the users in drawing and manipulation state than rest condition, which can be seen as control condition. From this figure, we can deduce that the information flow increase in the drawing and manipulation states compared to a rest state by establishing more connections. The main difference between novice and expert users was observed in drawing and manipulation states, that the CD relatively increased in manipulation states from drawing states for novice users (i.e., user 2–6). The expert users’ CD difference was much less compared to novices and a little decrease in density was seen for users 1, 7, and 8, which means that the information flow was approximately the same in drawing and manipulation states. The increase in connectivity for novice users also indicated that the electrodes were trying to establish a mutual connection to facilitate effective information transfer within the FBN as novice users are using the manipulation functions for the first time.

**Figure 11:**
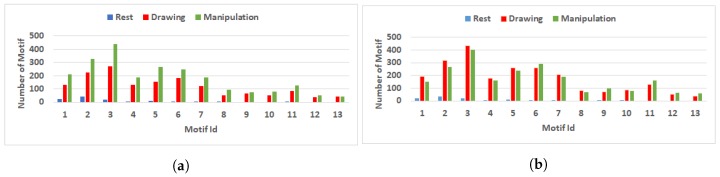
Comparison of number of motif with three nodes during rest, drawing and manipulation states. (**a**) User 3 (Novice user). (**b**) User 8 (Expert user).

Figure 11 shows the total number of motif for three nodes during rest, drawing and manipulation state for user 3 (novice) and user 8 (expert).

The motif count is higher in drawing and manipulation states compared to the rest state. Novice users have an increase in the number of motifs in manipulation states from drawing state and this pattern exists for almost all the novice users except for user 4, in which the motif count difference is not significant. Like CD, the motif count for expert users decreases in manipulation state compared to drawing state. As the number of motifs is used to describe the local features of the network, the increase in motifs is related to a substantial increase of information exchange among the neighboring nodes of directed FBN. The information transfer between neighbor electrodes was more for novice users than expert users, which means that novice user’s information flow pattern changes more rapidly compared to expert users.

**Figure 12:**
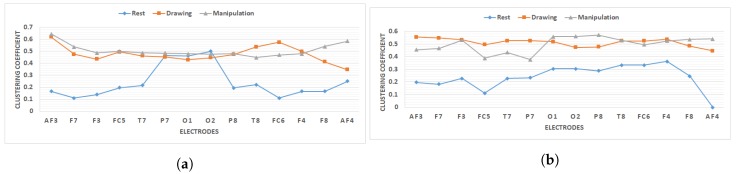
Clustering coefficient of all electrodes for novice and expert users during rest, drawing and manipulation states. (**a**) User 3 (A representative novice user). (**b**) User 8 (A representative expert user).

Clustering coefficient for all electrodes has been shown in Figure 12 during rest, drawing and manipulation states.

The value increases for almost all the electrodes in drawing and manipulation states compared to baseline rest state. This is a clear indication that each electrode was communicating directly with it is neighboring electrodes and formed clusters. For novice users, the clustering coefficient value in manipulation state is slightly higher than drawing state except for T8 and FC6 electrodes. The trend is opposite for experts, the value in drawing state is higher than manipulation state except for electrodes O1, O2, P3. These regions belonged to the occipital and parietal cortex and from the literature, we found that these regions were associated with sensation from muscles, visual perception, and recognition [39].

The statistical significance of the clustering coefficient across all 14 electrodes has been calculated using a 2-sample *t*-test with unequal variance at α=0.05 for novice and expert users. The results are shown in Table 2 for 2 typical users due to space limitations.

The results suggested that mean difference is significant (p<0.05) across rest/drawing and rest/manipulation states for both users and the same trend was observed for all other users. The difference is not significant across manipulation/drawing state but if we compare the values for novice and experts the difference across manipulation/drawing state is more for novice users than experts with a negative mean difference for user 8 and 9.

The small-world properties of directed FBN during rest, drawing and manipulation states for novice and expert users are shown in Table 3.

In Table 3, $C_{d}$ and $C_{rand}$ are the clustering coefficient of an actual and random network respectively; $L_{d}$ and $L_{rand}$ are the characteristic path lengths of actual network and random network, respectively. If the value of σ>1 for an FBN then the network shows the small-world properties which means that the FBN has both high local and global efficiency. For novice users the difference between σ in rest, drawing and manipulation states is more than expert, which means that experts have relatively high global and local efficiency of information transfer than novice users. To calculate the random values for clustering coefficient and characteristic path length, 100 matched random networks have been generated with 14 nodes. The small-world values are computed with transitive clustering coefficient and Monte-Carlo realizations [30].

### 4.2. Weighted FBN Analysis

**Figure 13:**
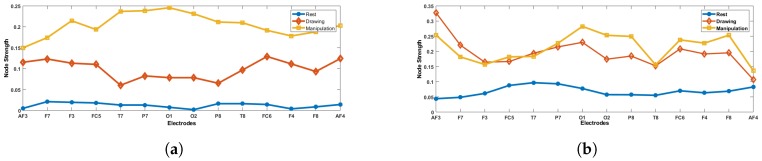
Comparison of node strength across electrodes during rest, drawing and manipulation states. (**a**) User 3 (Novice). (**b**) User 8 (Expert).

Figure 13 gives the node strength values of novice and expert users during various cognitive states. From Figure 13, we can observe that both novice and expert users have more node strength in drawing and manipulation states than the rest state, which indicates that each node or electrode sends and receives more information during drawing and manipulation states. The expert user’s node strength is approximately the same in drawing and manipulation states which means that their brain is more relaxed and information flow is the same in both states. Whereas, for novice users, the node strength increases in manipulation state, which indicates more information transfer in manipulation state than any other state. All the novice users showed the same trend of node strength.

#### 4.2.1. Statistical Analysis

**Figure 14:**
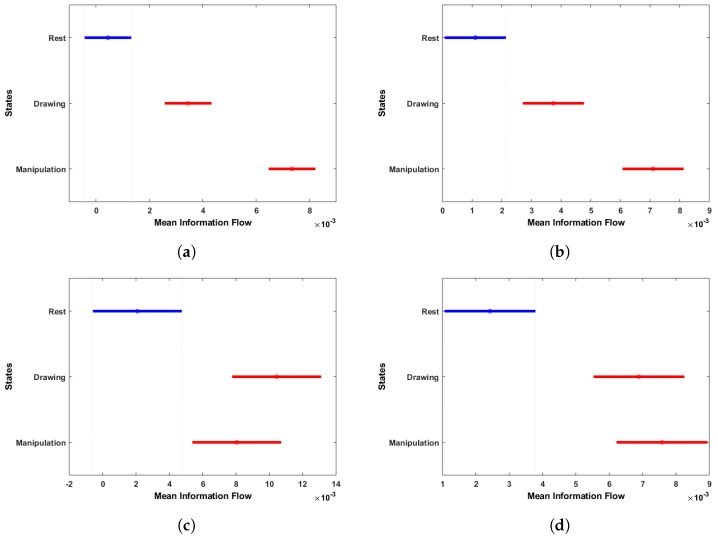
Multi-comparison of ANOVA on mean information flow of each electrode to all other electrodes during rest, drawing and manipulation states. (**a**) User 3 (Novice). (**b**) User 6 (Novice). (**c**) User 7 (Expert). (**d**) User 8 (Expert).

To show the significance of information flow during the three states, we have applied the one-way analysis of variance (ANOVA) test. The mean information flow of each state, which is calculated by row-wise summation of each NTE matrix, was used in ANOVA and the multi-comparison procedure was applied and results are shown in Figure 14.

The ANOVA results show that there are significant differences in the mean information flow between rest and drawing/manipulation states. For novice users, the mean information flow difference is also significant in drawing and manipulation states, but this is not the case for expert users because of the overlap seen in drawing and manipulation states mean information flow. This is a clear indication that the information flow increased in novice users, when they started manipulating the object, whereas expert users were comfortable with both drawing and manipulation.

#### 4.2.2. Hemisphere-Wise Analysis

**Figure 15:**
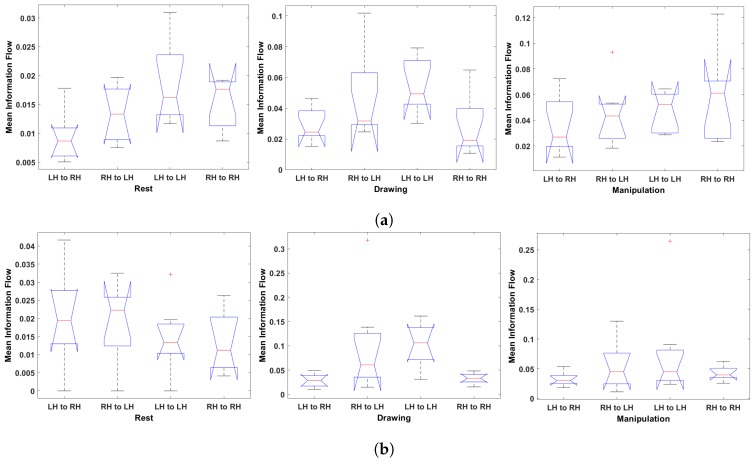

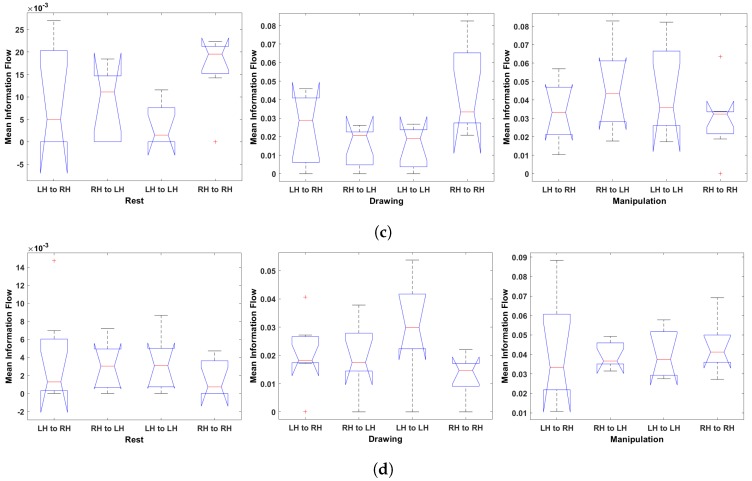
One-way ANOVA of Hemisphere-wise mean information flow during rest, drawing and manipulation states. (**a**) User 7 (Expert). (**b**) User 8 (Expert). (**c**) User 3 (Novice). (**d**) User 6 (Novice).

Figure 15a–d shows the ANOVA test results for hemisphere-wise analysis. The results show that the information transfer from LH to LH is greater than the information transfer in other hemispheres for expert users 7 and 8 in drawing state. In manipulation state, the *t*-test results showed that there are no significant differences in mean information transfer value for RH to RH and RH to LH transfer. For user 7, the values of RH to LH and RH to RH increased in manipulation state compared to drawing state. User 8 shows an opposite behavior, the mean information flow value from RH to LH and LH to LH decreases in manipulation state and other information transfer values do not change much. In case of the novice users, an increase in information flow in all the electrodes was observed from rest to drawing to manipulation state. The flow of information is more towards the left hemisphere from the right and left hemisphere electrodes. Thus, it can be said that, in manipulation state, the left region of the brain has received more information from its own electrodes then the right-side electrodes.

The statistical significance of the difference in information transfer between different states has been given in Table 4. These results were calculated by applying the two-sample *t*-test with α=0.05 (two-tailed). The results show that in most cases the difference in mean information flow is significant especially for novice users. The mean difference is more for novice users when comparing manipulation and drawing states. For experts, a small decrease in information flow was observed, when comparing the RH-LH and LH-LH regions in manipulation and drawing states. This is an indication that the flow of information decreases for experts in left hemisphere from left and right hemisphere electrodes. Overall, Novice users show significant mean differences in all three states compared to experts. With experts, the difference is not significant in all cases which indicates that the expert user’s brain trained in a way that it allows certain regions to be more active than others with respect to tasks.

#### 4.2.3. Region-Based Analysis

The region-based analysis of information flow is recorded in Table 5. The result shows that the information flow from F-C is high in experts compare to novice users and the IF from F-C is more than IF from C-F. The IF from F-C increased in manipulation state compared to other states for every user, which indicates that more information is transferred from the frontal region. The IF from P-C is more for novice users compared to expert users. The IF from F-P increases from rest to manipulation state for novice users but decreases for experts. From the analysis in manipulation state, maximum information was transferred through frontal electrodes. The reason for the maximum activation of the frontal scalp regions can be because this region is associated with reasoning, planning and problem solving [40].

In Table 5, the highlighted blue color shows an increase in information flow from drawing state and red color shows a decrease in IF. The analysis clearly shows that expert users IF increased in some region and decreased in other whereas for novice users the information flow has increased in manipulation state from drawing state with some exception (user 6). It is also an indication that expert users have developed the capability to control the performance of a specific brain region to perform a certain task effectively.

In rest state most of the IF is through central region and the least information transfer was in between frontal and parietal lobes for all users. All users show maximum information transfer from the frontal cortex in manipulation state and minimum IF has been seen in the central cortex in manipulation state. In drawing state maximum IF has been seen through the frontal to the central cortex. The parietal and occipital lobes have shown minimum activation in sending information except for user 8.

### 4.3. Classification Results

The classification results are shown in Table 6, Table 7 and Table 8. The data from all eight subjects were used in this experiment. The data set was divided into training and testing data-sets and the results are evaluated with 5-fold cross-validation. Table 6 shows the classification results of different classifiers with all feature as input. The LDA classifier shows maximum classification accuracy of 88% with an F-measure of 0.91. The NB classifier shows the least classification accuracy.

SFS has been used to select the best features and the results are shown in Table 7. The k-NN (k = 3) shows the best classification accuracy of 95% with just 11 features. The classification accuracy for all classifiers increased after applying SFS for feature selection except for LDA. NB classifier accuracy also increased from 69% to 78% by selecting only 4 features.

Table 8 shows the timing analysis to select five features using SFS. The SVM classifier takes the maximum time (818.47 s) and k-NN takes the least time (7.30 s) to select five features. k-NN also gives the maximum classification accuracy among all other algorithms. The results also show some common features selected by different classification algorithms. The feature number 29, 32, 35 and 41 selected by k-NN also appears in the features selected by other algorithms. The feature 29 is the characteristic path length and 32, 35, and 41 belong to the mean information flow. The feature 32, 35, and 41 correspond to the mean information flow of electrodes F3, T7 and F8, respectively. It shows that the channel that contributes to maximum variance between novice and expert users are F3, and F4 from frontal lobe and T8 from temporal lobe of the brain.

**Figure 16:**
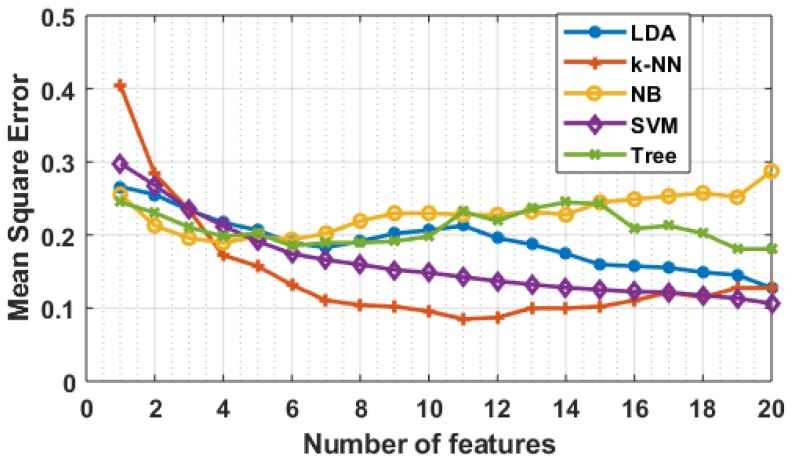
Mean square error with respect to features selected.

Figure 16 shows the mean square error (MSE) of various classification algorithm with respect to the features selected. The graph shows that the error decreased when the number of features increased. After selecting a certain number of features, the change in MSE was not significant. The least MSE of 8% was observed with k-NN classifier and NB shows the maximum MSE compared to other classifiers.

## 5. Conclusions

In this paper, a novice/expert study has been presented that uses NTE to construct FBN and estimate the information flow patterns. Both binary and weighted directed FBNs were used for the analysis. Using the techniques of signal and information processing to construct FBNs and applying graph theory and statistical analysis, we have estimated the cognitive load and information flow pattern for novice and expert users by measuring the CD, clustering coefficient, characteristic path length, motif count, node strength and small-worldness. The results showed a significant difference in information processing between novice and experts. The findings from the analysis are listed below:

1. The main difference was observed in the manipulation state, novice users information flow patterns changed in manipulation state compared to experts. The CD, motif count, and clustering coefficient all showed the same trend.
2. The network density increased from the baseline for both novice and expert users, but the change is more for novice users compared to experts in drawing and manipulation states.
3. Most of the activity was focused in the frontal region, which indicates the use of short-term memory.
4. The small-worldness shows that experts have relatively high global and local efficiency of information transfer than novices.
5. The hemisphere analysis shows that the information flow has increased in both hemispheres for novice users, but experts managed to control the information flow according to the task.
6. In the lobe-wise analysis, the frontal lobe was most active in sending and receiving information in drawing and manipulation states for all users.

Expert users have developed a capability which gives them control over different brain regions for different tasks, unlike novice users where almost all regions became active. The results indicate that experts were more relaxed in both drawing and manipulation states, whereas novice users put more effort into manipulation state than drawing state. If we somehow develop a system that allows the user to control the activation of different brain regions such as trans-cranial magnetic stimulation, then we may be able to decrease the novice user training time to become an expert. The results are aligned with the earlier findings of Kavalki & Gero [41] regarding differences between novice and experts concurrent cognitive processing.

The classification result shows the effectiveness of the proposed algorithm. A classification accuracy of more than 90% was achieved with the proposed technique using a simple k-NN classifier. The feature selection algorithm also helps us to find the features that maximize the accuracy. These features belong to the frontal and temporal lobes of the brain.

In the future, we will focus on using these differences for identifying novice and expert users in real time and develop an adaptive system for 3D modelling applications that will accommodate the differences in skill levels.

## Figures and Tables

**Table 1:** Time taken by each participant to complete the task.

Expertise	Users	Drawing (s)	Manipulation (s)
Expert	User 1	38	72
Novice	User 2	81	125
Novice	User 3	137	93
Novice	User 4	54	72
Novice	User 5	43	118
Novice	User 6	61	98
Expert	User 7	120	101
Expert	User 8	58	113

**Table 2:** Statistical validation of clustering coefficients for typical novice and expert users.

Users	States		Mean Difference	95% CI	DF	*t*	*p*
User 3 (Novice)	Drawing	Rest	0.156 *	(0.0763, 0.2357)	20	4.0838	0.0006
	Manipulation	Drawing	0.031	(−0.0169, 0.0789)	24	1.3362	0.097
	Manipulation	Rest	0.187 *	(0.1102, 0.2638)	17	5.1374	0.00001
User 8 (Expert)	Drawing	Rest	0.273 *	(0.2148, 0.3312)	15	10	0.00001
	Manipulation	Drawing	−0.015	(−0.0988, 0.0688)	17	−0.7895	0.3551
	Manipulation	Rest	0.258 *	(0.1935, 0.3225)	22	8.2958	0.00001

* Mean difference is significant at p<0.05 level.

**Table 3:** Small-worldness of binary directed FBNs during rest, drawing and manipulation states for 2 typical users.

Users	Cognitive State	$C_{d}$	$C_{rand}$	$L_{d}$	$L_{rand}$	$\sigma =\frac{C_{d}}{C_{rand}}/\frac{L_{d}}{L_{rand}}$
User 3 (Novice)	Rest	0.9119	0.8676	1.1939	1.1934	1.0506
	Drawing	0.6675	0.5358	1.5255	1.5043	**1.2286**
	Manipulation	0.7714	0.6582	1.3878	1.3879	1.0809
User 8 (Expert)	Rest	0.8214	0.6998	1.3469	1.3470	1.1739
	Drawing	0.4777	0.4126	1.7092	1.6537	**1.1202**
	Manipulation	0.6607	0.5267	1.5408	1.5256	**1.2420**

The bold number shows that σ>1.1.

**Table 4:** Result of pairwise mean difference using the *t*-test for hemispheres information flow during rest, drawing and manipulation states.

States		Novice User 3			Novice User 6		
Drawing	Rest	Mean Diff.	CI		Mean Diff.	CI	
LH-RH	LH-RH	0.017 *	0.005	0.029	0.016	−0.003	0.034
RH-LH	RH-LH	0.017 *	0.006	0.028	0.005	−0.004	0.019
LH-LH	LH-LH	0.027 *	0.011	0.043	0.012 *	0.001	0.022
RH-RH	RH-RH	0.012 *	0.005	0.018	0.030 *	0.007	0.052
**Manipulation**	**Drawing**						
LH-RH	LH-RH	0.022	−0.004	0.048	0.008	−0.014	0.029
RH-LH	RH-LH	0.020 *	0.008	0.032	0.031 *	0.001	0.052
LH-LH	LH-LH	0.010	−0.007	0.028	0.029 *	0.006	0.052
RH-RH	RH-RH	0.031 *	0.018	0.044	−0.016	−0.042	0.009
**Manipulation**	**Rest**						
LH-RH	LH-RH	0.039 *	0.014	0.064	0.024 *	0.002	0.041
RH-LH	RH-LH	0.037 *	0.01	0.043	0.038	0.017	0.059
LH-LH	LH-LH	0.037 *	0.026	0.048	0.041 *	0.018	0.064
RH-RH	RH-RH	0.043 *	0.030	0.055	0.014	−0.004	0.032
		**Expert User 7**			**Expert User 8**		
**Drawing**	**Rest**	**Mean Diff.**	**CI**		**Mean Diff.**	**CI**	
LH-RH	LH-RH	0.008	−0.008	0.024	0.020 *	0.009	0.030
RH-LH	RH-LH	0.080	−0.017	0.177	0.034 *	0.008	0.060
LH-LH	LH-LH	0.088 *	0.046	0.130	0.035 *	0.018	0.052
RH-RH	RH-RH	0.020 *	0.008	0.031	0.013	−0.005	0.031
**Manipulation**	**Drawing**						
LH-RH	LH-RH	0.005	−0.010	0.019	0.006	−0.016	0.028
RH-LH	RH-LH	−0.042	−0.141	0.057	−0.003	−0.035	0.028
LH-LH	LH-LH	−0.025	−0.108	0.057	−0.007	−0.027	0.013
RH-RH	RH-RH	0.009	−0.005	0.023	0.029	−0.005	0.063
**Manipulation**	**Rest**						
LH-RH	LH-RH	0.013	−0.002	0.027	0.026 *	0.004	0.047
RH-LH	RH-LH	0.038	−0.000	0.076	0.031 *	0.008	0.054
LH-LH	LH-LH	0.063	−0.017	0.142	0.028 *	0.013	0.043
RH-RH	RH-RH	0.029 *	0.016	0.041	0.042 *	0.009	0.075

* Mean difference is significant at p<0.05 level.

**Table 5:** Information flow among F, C and P nodes during rest, drawing and manipulation states.

Subjects	States	F-C	C-F	F-P	P-F	P-C	C-P	Max	Min
**User 3 (Novice)**	**Rest**	0.0061	0.0121	0.0111	0.0037	0.0048	0.0058	C-F	P-F
	**Drawing**	0.0611	0.0502	0.0709	0.0224	0.0330	0.0637	F-P	P-F
	**Manipulation**	**0.1097**	**0.0919**	**0.1350**	**0.1091**	**0.0987**	**0.1139**	F-P	C-F
**User 6 (Novice)**	**Rest**	0.0212	0.0162	0.0135	0.0313	0.0372	0.0242	P-C	F-P
	**Drawing**	0.0741	0.0555	0.0434	0.0386	0.0964	0.0730	P-C	P-F
	**Manipulation**	**0.1080**	**0.0797**	**0.0557**	**0.0579**	**0.0431**	**0.0636**	F-C	P-C
**User 8 (Expert)**	**Rest**	0.0465	0.0325	0.0175	0.0254	0.0359	0.0446	F-C	F-P
	**Drawing**	0.0736	0.0702	0.1154	0.1516	0.0622	0.0606	P-F	C-P
	**Manipulation**	**0.1792**	**0.1215**	**0.1027**	**0.1478**	**0.0526**	**0.1020**	F-C	P-C
**User 7 (Expert)**	**Rest**	0.0242	0.0134	0.0356	0.0406	0.0606	0.0689	C-P	C-F
	**Drawing**	0.2260	0.0752	0.1570	0.0739	0.0735	0.1027	F-C	P-C
	**Manipulation**	**0.1631**	**0.0577**	**0.0952**	**0.0642**	**0.0782**	**0.0590**	F-C	C-F

**Table 6:** Classification results using all features.

Classifiers	Acc	Sen	Spec	F-Measure
**LDA**	88%	0.94	0.80	0.91
**KNN**	71%	0.75	0.65	0.76
**NB**	69%	0.80	0.53	0.76
**SVM**	82%	0.87	0.74	0.85
**Tree**	83%	0.88	0.75	0.86

**Table 7:** Classification results after feature selection by SFS.

Classifiers	Acc	Sen	Spec	F-Measure	Features
**LDA**	88%	0.95	0.76	0.90	40.00
**KNN**	95%	0.96	0.92	0.96	11.00
**NB**	78%	0.93	0.56	0.84	4.00
**SVM**	88%	0.95	0.76	0.90	20.00
**Tree**	89%	0.90	0.88	0.91	16.00

**Table 8:** Time taken by the algorithm to select features using various classifiers.

Classifiers	Acc	Feature Selected	Time in s
**LDA**	80%	12 32 35 37 41	7.96
**KNN**	92%	23 29 32 35 41	7.30
**NB**	78%	29 30 32 35 41	7.52
**SVM**	81%	19 20 29 32 38	818.47
**Tree**	86%	9 17 22 29 35	7.71
